# Governing algorithms, empowering people: how ethical AI oversight shapes trust, technostress, and employee autonomy in AI-enabled HRM

**DOI:** 10.3389/frai.2026.1911545

**Published:** 2026-08-31

**Authors:** Tamrisha Patnaik, Robert Ipiin Gnankob, Nitya Sundar Nanda

**Affiliations:** 1 School of Business, Woxsen University, Hyderabad, India; 2 Vignana Jyothi Institute of Management, Hyderabad, India

**Keywords:** AI in HRM, algorithmic management, employee autonomy, PLS-SEM, technostress

## Abstract

**Introduction:**

Artificial intelligence is increasingly embedded in human resource management, yet its implications for employee autonomy depend not only on technological capability but also on how algorithmic systems are governed. This study examines how algorithmic transparency, perceived algorithmic fairness, and human-in-the-loop oversight shape employee autonomy through trust in AI, psychological safety, and technostress.

**Methods:**

Drawing on Social Exchange Theory and Conservation of Resources Theory, the study tested an integrated model using survey data from 290 employees working in organisations that employ AI-enabled HRM systems in India. The hypothesised direct and mediated relationships were examined using partial least squares structural equation modelling.

**Results:**

Algorithmic transparency, perceived algorithmic fairness, and human-in-the-loop oversight significantly strengthened employee trust in AI. Algorithmic transparency also enhanced psychological safety, while human-in-the-loop oversight reduced technostress. Trust in AI and psychological safety positively influenced employee autonomy, whereas technostress negatively influenced autonomy. Trust mediated the relationships between transparency, fairness, human oversight, and employee autonomy. Psychological safety mediated the transparency–autonomy relationship, while technostress mediated the relationship between human oversight and autonomy.

**Discussion:**

The findings demonstrate that employee autonomy in AI-enabled workplaces is shaped through relational, psychological, and resource-preservation mechanisms. Responsible AI-enabled HRM therefore requires more than technical efficiency; transparent decision processes, fairness safeguards, meaningful human oversight, and psychologically safe opportunities to question algorithmic decisions are important for preserving employee agency.

## 1 Introduction

Artificial intelligence (AI) is increasingly transforming how organisations recruit, evaluate, schedule, develop, reward, and retain employees. Algorithms now screen résumés, recommend candidates, predict employee turnover, allocate tasks, monitor performance, and inform promotion and compensation decisions. These developments extend beyond the automation of routine administrative activities. By influencing how employment decisions are generated and implemented, AI-enabled human resource management (HRM) systems increasingly perform functions previously exercised through managerial judgement. This emerging form of organisational control is commonly described as algorithmic management: the coordination, evaluation, and regulation of work through data-driven systems and software algorithms (Duggan et al., 2020; Kellogg et al., 2020; Meijerink and Bondarouk, 2023).

The adoption of AI in HRM offers considerable organisational benefits. Algorithmic systems can process large volumes of workforce data, identify patterns that human decision-makers may overlook, improve the consistency of routine decisions, and support more responsive workforce planning. Recent reviews show that AI applications are now used across recruitment, performance evaluation, talent management, employee development, workforce planning, and compensation management (Malik et al., 2023; Bujold et al., 2024). Nevertheless, the use of AI to manage people also introduces substantial ethical and organisational risks. Algorithms may reproduce biases embedded in historical data, operate through decision rules that employees cannot understand, intensify workplace surveillance, and obscure responsibility when decisions produce unfair outcomes (Barocas et al., 2019; Wieringa, 2020; Bujold et al., 2024). Responsible AI in HRM therefore requires more than technical accuracy; it also demands transparency, fairness, accountability, employee voice, and continued human involvement in consequential decisions.

These concerns are particularly important because HR decisions directly affect employees’ careers, livelihoods, and everyday work experiences. When performance ratings, promotion recommendations, schedules, or disciplinary decisions are generated through opaque systems, employees may struggle to understand how they were assessed or how an unfavourable outcome can be challenged. Such information asymmetry can create helplessness and reduce employees’ perceived influence over decisions that affect them (Lee, 2018). Although algorithms are often promoted as more consistent and objective than human decision-makers, apparent neutrality does not necessarily guarantee fairness. Algorithmic systems can reinforce existing inequalities, apply criteria without sufficient contextual sensitivity, and generate outcomes that employees perceive as arbitrary or unjust (Binns et al., 2018; Grgic-Hlaca et al., 2018; Barocas et al., 2019).

A central outcome implicated in these developments is employee autonomy. Employee autonomy refers to the degree to which individuals experience discretion, independence, and control in performing their work (Hackman and Oldham, 1976). It is also closely associated with the psychological need for self-direction and volitional action (Deci and Ryan, 2000). Autonomy supports intrinsic motivation, creativity, psychological well-being, adaptability, and organisational commitment. Yet algorithmic management may threaten autonomy when software systems prescribe how work should be undertaken, continuously monitor employee behaviour, standardise decisions, or substitute automated instructions for contextual human judgement (Kellogg et al., 2020).

The relationship between algorithmic management and autonomy is, however, not uniformly negative. Algorithms may constrain employees through monitoring and standardisation, but they may also enable autonomy by providing useful information, reducing routine workloads, coordinating complex tasks, and allowing greater flexibility in organising work. Meijerink and Bondarouk (2023) describe this as the duality of algorithmic management: the same technological features may simultaneously enable and restrict employee autonomy. Similarly, recent research suggests that algorithmic management can reproduce digitally intensified forms of control while also creating opportunities for flexibility and self-direction, depending on how the technology is configured and governed (Noponen et al., 2024). The crucial issue is therefore not simply whether organisations adopt AI-enabled HRM, but whether the governance of such systems preserves meaningful employee agency.

This study focuses on three governance mechanisms that may determine whether AI-enabled HRM supports or undermines employee autonomy: algorithmic transparency, perceived algorithmic fairness, and human-in-the-loop presence. Algorithmic transparency concerns the extent to which employees receive understandable information about the data, criteria, and reasoning underlying algorithmic decisions (Lepri et al., 2018). When employees understand how performance scores, schedules, or recommendations are generated, they may be better positioned to interpret, question, and respond to those decisions (Lee, 2018). Perceived algorithmic fairness refers to employees’ assessments of whether algorithm-supported HR processes and outcomes are consistent, unbiased, appropriate, and just. Organisational justice research has long shown that employees evaluate both the procedures used to make decisions and the outcomes produced by those procedures (Greenberg, 1987). These evaluations remain important when decision authority is delegated to AI (Binns et al., 2018; Bujold et al., 2024).

Human-in-the-loop presence represents the meaningful retention of human judgement and accountability within AI-supported decision processes (Cummings, 2004; Amershi et al., 2019). In HRM, human involvement may allow managers to review, interpret, revise, or overturn algorithmic recommendations. It may also provide employees with an identifiable point of contact through whom they can seek clarification, correct inaccurate information, or appeal an unfavourable decision. This is important because employees may prefer imperfect human judgement to algorithmic decisions that appear unchallengeable or insensitive to context, reflecting the broader phenomenon of algorithm aversion (Dietvorst et al., 2016). Human oversight may therefore have psychological significance beyond its technical function by signalling accountability and restoring a sense of recourse.

The effects of these governance mechanisms on autonomy are unlikely to be exclusively direct. Employees interpret AI governance through psychological and relational processes. Trust in AI is one such process and reflects employees’ beliefs that an AI system is competent, reliable, predictable, and appropriate for the decisions it supports (Heerink et al., 2010). Transparent explanations, fair procedures, and visible human accountability may strengthen trust by reducing uncertainty and signalling responsible organisational conduct. Psychological safety may provide a second pathway. It reflects the perception that individuals can speak up, ask questions, and raise concerns without fear of punishment or humiliation (Edmondson, 1999). In algorithmically managed workplaces, employees must feel safe to challenge inaccurate data, report bias, or request human reconsideration.

Technostress represents a contrasting resource-depletion pathway. It describes the strain arising from individuals’ inability to cope effectively with new or demanding technologies and may include techno-overload, techno-complexity, techno-invasion, and techno-insecurity (Brod, 1984; Tarafdar et al., 2007; Ragu-Nathan et al., 2008). Employees exposed to algorithmic monitoring or automated evaluation may experience stress when they do not understand how decisions are made or believe they have little control over the system. Meaningful human oversight may reduce such strain by providing clarification, intervention, and opportunities for appeal.

Despite growing interest in responsible AI, these governance and psychological mechanisms remain insufficiently integrated in empirical AI-HRM research. Existing studies have often examined transparency, fairness, trust, technostress, psychological safety, and autonomy in separate streams. Recent reviews also indicate that responsible AI-HRM research remains concentrated in recruitment, selection, laboratory experiments, and hypothetical scenarios, with limited field evidence on how governance arrangements jointly shape employee experiences in actual organisations (Malik et al., 2023; Bujold et al., 2024; Noponen et al., 2024).

Against this background, the study examines how algorithmic transparency, perceived algorithmic fairness, and human-in-the-loop presence influence employee autonomy through trust in AI, psychological safety, and technostress. Drawing on Social Exchange Theory and Conservation of Resources Theory, it makes three contributions. First, it develops an integrated framework linking distinct ethical AI-governance mechanisms to employee autonomy. Second, it identifies three complementary pathways: a relational pathway through trust in AI, an interpersonal-risk pathway through psychological safety, and a resource-preservation pathway through reduced technostress. Third, it positions employee autonomy as an important criterion for evaluating responsible AI-enabled HRM. Responsible AI should therefore be assessed not only by efficiency, accuracy, or adoption, but also by whether AI-supported systems preserve employees’ discretion, voice, dignity, and meaningful control over their work.

## 2 Literature review and hypotheses

### 2.1 Theoretical underpinning

This study is anchored in social exchange theory and conservation of resources theory, which together explain how employees interpret and respond to AI-enabled HRM systems. Social Exchange Theory holds that workplace relationships develop through reciprocal exchanges in which employees evaluate whether organisational practices communicate fairness, support, respect, and concern for their interests (Blau, 1964; Cropanzano and Mitchell, 2005). In AI-enabled workplaces, employees are likely to interpret algorithmic transparency, perceived fairness, and human oversight as signals of how responsibly the organisation exercises technological control. Transparent decision processes reduce informational asymmetry, fairness communicates procedural respect, and human-in-the-loop presence demonstrates that the organisation remains accountable for decisions generated through AI. These governance practices may therefore strengthen trust in AI and create an environment in which employees feel safer questioning, interpreting, or challenging algorithmic outcomes.

Conservation of resources theory complements this relational explanation by focusing on the psychological resources employees seek to acquire and protect, including control, certainty, time, energy, knowledge, and emotional security (Hobfoll et al., 2018). AI-enabled HRM may threaten these resources when employees are continuously monitored, evaluated through opaque criteria, or required to respond to automated decisions they cannot influence. Such conditions can generate technostress through complexity, insecurity, overload, and perceived loss of control (Tarafdar et al., 2007; Ragu-Nathan et al., 2008). Meaningful human oversight may reduce this resource depletion by providing explanation, contextual judgement, correction, and opportunities for appeal. Psychological safety similarly provides an interpersonal resource that allows employees to raise concerns about AI decisions without fearing embarrassment, punishment, or professional disadvantage (Edmondson, 1999).

Given the theoretical lenses, we deduce that, first, transparency, fairness, and human accountability may generate trust by signalling responsible organisational conduct. Second, transparent governance may strengthen psychological safety by making algorithmic decisions more understandable and contestable. Third, human oversight may reduce technostress by protecting employees from uncertainty and uncontestable technological control. Trust and psychological safety may consequently support employees’ willingness and confidence to exercise judgement, while reduced technostress preserves the cognitive and emotional resources required for autonomous action. This integrated theoretical position is consistent with the dual nature of algorithmic management, which may either enable or restrict autonomy depending on how AI systems are designed and governed (Kellogg et al., 2020; Meijerink and Bondarouk, 2023).

### 2.2 Ethical AI-governance mechanisms and employee trust in AI

Employee trust in AI refers to the belief that an AI system is competent, reliable, predictable, and appropriate for the organisational decisions it supports (Heerink et al., 2010). Trust is particularly important in AI-enabled HRM because employees are affected by systems that may evaluate performance, recommend promotion, determine schedules, or influence recruitment and disciplinary decisions. Although technical accuracy may contribute to trust, employees also assess whether the organisation governs the system transparently, fairly, and accountably. Accordingly, trust in AI is not simply a reaction to technological performance; it also reflects employees’ interpretation of the governance arrangements surrounding the technology.

Algorithmic transparency concerns the extent to which employees receive understandable information about the data, criteria, processes, and reasoning used in algorithm-supported decisions (Lepri et al., 2018). Transparency can strengthen trust by reducing the information asymmetry between organisations and employees. When employees understand why an algorithm produced a particular recommendation or outcome, they are better able to evaluate its consistency, relevance, and legitimacy. Transparency also enhances predictability because employees can anticipate how their actions or performance may be assessed. In contrast, opaque systems may generate uncertainty and suspicion, particularly when employees cannot determine whether a decision resulted from relevant information, inaccurate data, or hidden criteria (Lee, 2018; Wieringa, 2020).

Recent evidence reinforces the trust-building potential of transparency in algorithmically managed workplaces. Transparency in algorithmic performance management has been associated with more constructive employee responses because it clarifies performance expectations and the processes through which algorithmic evaluations are produced. However, transparency must be meaningful and understandable rather than consisting merely of technical disclosure that employees cannot interpret. In responsible AI-enabled HRM, employees require explanations that allow them to assess how the system operates and how its outcomes affect them (Bujold et al., 2024; Cai and Wang, 2024). Therefore, when organisations explain their algorithmic decision processes clearly, employees are more likely to regard AI-supported systems as predictable and trustworthy. Accordingly:

*H1:* Algorithmic transparency is positively related to employee trust in AI.

Perceived algorithmic fairness refers to employees’ judgement that algorithmic procedures and outcomes are consistent, unbiased, equitable, and based on appropriate information. Organisational justice research establishes that employees evaluate both how decisions are made and how resulting outcomes are distributed (Greenberg, 1987). These evaluations remain important when AI participates in organisational decision-making. Employees may distrust an algorithm that appears to reproduce historical bias, applies inappropriate criteria, ignores relevant contextual information, or generates outcomes that disadvantage particular individuals or groups (Binns et al., 2018; Grgic-Hlaca et al., 2018; Barocas et al., 2019).

Fairness perceptions are particularly salient in HRM because employment decisions have material and symbolic consequences. An employee who regards an algorithmic performance rating or promotion recommendation as unfair may question not only the system’s competence but also the intentions of the organisation that adopted it. Conversely, perceptions that AI-supported procedures are impartial and consistently applied can signal that the organisation is committed to responsible and respectful treatment. Recent research on AI-enabled recruitment similarly positions procedural, distributive, interpersonal, and informational justice as important foundations of trust and acceptance (Liu et al., 2023). Research also shows that replacing human decision-makers with AI can alter fairness perceptions, particularly where individuals believe that contextual judgement and human sensitivity are necessary (Köchling et al., 2025).

Perceived fairness is therefore expected to increase employees’ confidence that AI-supported HR decisions are legitimate and dependable. When employees believe that algorithmic systems use relevant information, apply standards consistently, and do not systematically disadvantage particular groups, they should be more willing to trust those systems. Accordingly:

*H2:* Perceived algorithmic fairness is positively related to employee trust in AI.

Human-in-the-loop presence refers to the meaningful retention of human judgement and accountability within AI-supported decision processes (Cummings, 2004; Amershi et al., 2019). In HRM, this means that managers do not merely implement algorithmic recommendations automatically but retain the authority and responsibility to review, interpret, revise, or overturn them. Human oversight may also provide employees with opportunities to request explanations, correct inaccurate information, and appeal decisions that they consider inappropriate.

The presence of accountable human decision-makers can strengthen trust in AI in several ways. First, human oversight may compensate for employees’ concerns that algorithms cannot fully appreciate contextual, relational, or exceptional circumstances. Second, it creates a visible point of responsibility when algorithmic recommendations produce errors or harmful outcomes. Third, it signals that AI is intended to support rather than completely replace managerial judgement. This distinction is important because employees may resist systems they perceive as exercising autonomous and uncontestable authority, even when those systems are technically capable. Algorithm aversion research similarly shows that individuals often prefer systems that permit human adjustment rather than algorithms whose outputs cannot be modified (Dietvorst et al., 2016).

Recent research on digital HRM further indicates that human decision-makers remain important to the quality and legitimacy of AI-supported personnel selection. Human involvement can improve contextual interpretation, although its value depends on whether decision-makers critically examine rather than automatically follow algorithmic recommendations. Contemporary human–AI collaboration research therefore increasingly argues that humans should remain active participants with genuine decision authority, rather than functioning as symbolic reviewers of machine-generated outputs. When employees recognise that a responsible person can examine and correct algorithmic decisions, they are likely to perceive the system as more accountable and trustworthy. Accordingly:

*H3:* Human-in-the-loop presence is positively related to employee trust in AI.

### 2.3 Algorithmic transparency and psychological safety

Psychological safety refers to employees’ shared or individual perception that they can express concerns, ask questions, acknowledge uncertainty, and challenge prevailing practices without fear of embarrassment, retaliation, or professional harm (Edmondson, 1999). In AI-enabled HRM, psychological safety becomes particularly important because employees may need to question algorithmic recommendations, report inaccurate data, identify possible bias, or request reconsideration of automated decisions. The presence of formal appeal procedures alone may be insufficient where employees do not feel safe to use them.

Algorithmic transparency can strengthen psychological safety by reducing the ambiguity surrounding AI-supported decisions. When employees understand the information used by an algorithm, the criteria applied, and the reasoning behind a recommendation, they are better positioned to evaluate the outcome and articulate specific concerns. Transparency therefore gives employees an informational basis for voice. Rather than challenging an apparently inscrutable system, employees can identify where data may be incomplete, assumptions may be inappropriate, or decision rules may have been applied incorrectly (Lee, 2018; Lepri et al., 2018; Wieringa, 2020).

Transparency may also communicate that organisational decision processes are open to scrutiny. When organisations explain how AI systems operate and acknowledge their limitations, they signal that algorithmic outcomes are not beyond discussion or correction. Such openness can reduce the interpersonal risk associated with questioning technology-supported decisions. Conversely, opaque systems may discourage employee voice because workers cannot determine whether their concerns are legitimate, whether managers understand the system, or whether challenging an algorithm will be interpreted as resistance to organisational innovation.

This relationship is especially relevant in HRM because algorithmic decisions can affect performance evaluations, promotion opportunities, work allocation, and disciplinary outcomes. Recent research on responsible AI in HRM emphasises that explainability, stakeholder participation, opportunities for appeal, and human accountability are essential for creating legitimate and employee-centred AI systems (Bujold et al., 2024). Transparency can therefore contribute to psychological safety when it enables employees not only to understand AI-supported decisions but also to engage constructively with them.

From a social exchange theory perspective, transparent governance signals respect, openness, and organisational willingness to remain accountable. Employees who receive understandable explanations may infer that the organisation values their right to know how decisions affecting them are made. This favourable relational signal should increase their confidence that questions or objections will be treated as legitimate rather than punished. Accordingly:

*H4:* Algorithmic transparency is positively related to employee psychological safety.

### 2.4 Human-in-the-loop presence and technostress

Technostress refers to the psychological strain employees experience when technological demands exceed their perceived capacity to understand, manage, or control them (Brod, 1984; Tarafdar et al., 2007; Ragu-Nathan et al., 2008). In AI-enabled HRM, technostress may arise when employees are continuously monitored, evaluated through opaque systems, or subjected to automated recommendations whose logic and consequences are difficult to interpret. These conditions can create techno-complexity, insecurity, overload, and a diminished sense of control, particularly when AI-generated decisions affect performance ratings, work allocation, promotion, or disciplinary outcomes.

Human-in-the-loop presence may reduce these pressures by preserving meaningful human judgement within algorithmic decision processes. When managers retain the authority to interpret, revise, or overturn AI-generated recommendations, employees are less likely to perceive the system as autonomous and uncontestable. Human oversight also provides an identifiable source of explanation and recourse, allowing employees to clarify how a decision was reached, correct inaccurate information, and seek reconsideration where contextual factors have been overlooked (Cummings, 2004; Amershi et al., 2019).

From a conservation of resources perspective, uncontestable algorithmic control threatens valuable employee resources such as certainty, control, confidence, and psychological security (Hobfoll, 1989; Hobfoll et al., 2018). Employees may expend substantial cognitive and emotional resources trying to understand unfamiliar systems or anticipate how automated evaluations will affect them. Human oversight can offset this resource loss by providing reassurance, contextual interpretation, and opportunities for intervention. It therefore functions not only as a technical safeguard but also as a psychological resource that reduces uncertainty and perceived helplessness.

The effectiveness of human oversight, however, depends on whether it is meaningful rather than symbolic. A nominal reviewer who simply endorses algorithmic outputs may do little to reduce employee anxiety. By contrast, genuine human involvement can signal that employees are not entirely subject to automated control. Recent responsible AI scholarship similarly emphasises that human supervision should involve real authority, reflection, and intervention rather than procedural compliance alone (Bujold et al., 2024). Accordingly:

*H5:* Human-in-the-loop presence is negatively related to employee technostress.

### 2.5 Trust in AI, technostress, psychological safety, and employee autonomy

Employee autonomy reflects the extent to which employees experience discretion, independence, and control over how they perform their work and make work-related decisions (Hackman and Oldham, 1976). In AI-enabled workplaces, autonomy does not necessarily imply freedom from technological support. Rather, it concerns whether employees retain meaningful judgement and influence while interacting with algorithmic systems. Trust in AI, technostress, and psychological safety are therefore expected to shape whether employees experience AI as enabling or controlling.

Trust in AI may strengthen employee autonomy by increasing employees’ confidence in the reliability and legitimacy of AI-supported decisions. When employees believe that an AI system is competent, predictable, and appropriately governed, they are less likely to devote attention to questioning its basic reliability or protecting themselves against possible errors. Trust can therefore reduce defensiveness and allow employees to engage with AI-generated information as a supportive resource rather than an external source of control. From a Social Exchange Theory perspective, trust also reflects employees’ favourable interpretation of the organisation’s use of AI. Employees who perceive the system as fair and accountable may feel more secure in exercising judgement within the boundaries of AI-supported work. Conversely, distrust may encourage avoidance, resistance, excessive compliance, or dependence on managerial reassurance, all of which may weaken perceived autonomy. Accordingly:

*H6:* Employee trust in AI is positively related to employee autonomy.

Technostress is expected to have the opposite effect. When employees experience algorithmic systems as complex, intrusive, unpredictable, or difficult to control, they may expend considerable cognitive and emotional resources trying to understand system requirements and anticipate their consequences. Such resource depletion can narrow attention, reduce confidence, and encourage rigid compliance with algorithmic instructions. Conservation of Resources Theory suggests that employees become more protective and less willing to exercise discretion when valuable resources such as energy, certainty, control, and psychological security are threatened (Hobfoll, 1989; Hobfoll et al., 2018). Technostress may therefore undermine autonomy by reducing employees’ capacity and willingness to make independent work-related decisions. Accordingly:

*H7:* Technostress is negatively related to employee autonomy.

Psychological safety may also enhance autonomy by creating an environment in which employees can exercise judgement without fearing interpersonal or organisational consequences. In AI-enabled HRM, autonomy requires more than formal discretion; employees must feel able to question inaccurate outputs, report bias, request clarification, and propose alternatives to algorithmic recommendations. When employees perceive that such actions are legitimate and safe, they are more likely to participate actively in decisions rather than defer automatically to the system. Psychological safety therefore supports the voice, confidence, and interpersonal risk-taking needed for meaningful employee agency (Edmondson, 1999). In contrast, employees who fear retaliation or embarrassment may remain silent even when they recognise errors or inappropriate algorithmic outcomes, thereby allowing the system to constrain their effective autonomy. Accordingly:

*H8:* Psychological safety is positively related to employee autonomy.

### 2.6 Mediating roles of trust in AI, psychological safety, and technostress

The preceding arguments suggest that ethical AI-governance mechanisms influence employee autonomy not only directly, but also through employees’ relational and psychological responses to algorithmic management. Trust in AI is expected to transmit the effects of algorithmic transparency, perceived fairness, and human-in-the-loop presence to autonomy. Transparency reduces uncertainty, fairness strengthens perceptions of legitimacy, and human oversight signals accountability. These governance conditions should increase employees’ confidence that AI-supported HR systems are reliable and appropriately managed. In turn, employees who trust such systems are more likely to engage with them constructively while retaining confidence in their own judgement. Trust therefore provides the relational mechanism through which responsible AI governance supports employee autonomy. Accordingly:

*H9a:* Employee trust in AI mediates the positive relationship between algorithmic transparency and employee autonomy.

*H9b:* Employee trust in AI mediates the positive relationship between perceived algorithmic fairness and employee autonomy.

*H9c:* Employee trust in AI mediates the positive relationship between human-in-the-loop presence and employee autonomy.

Psychological safety is also expected to mediate the relationship between algorithmic transparency and autonomy. Transparency gives employees access to the information required to understand and evaluate algorithmic decisions. However, access to information does not automatically produce meaningful employee agency. Employees must also feel able to question decisions, identify errors, and express concerns without fear of retaliation. By signalling openness and organisational willingness to subject algorithmic decisions to scrutiny, transparency may strengthen psychological safety. Psychological safety, in turn, enables employees to exercise voice and judgement when engaging with AI-supported HR processes. Accordingly:

*H10:* Psychological safety mediates the positive relationship between algorithmic transparency and employee autonomy.

Technostress is expected to mediate the relationship between human-in-the-loop presence and employee autonomy. Human oversight provides explanation, contextual interpretation, and opportunities for correction or appeal, thereby reducing employees’ sense that algorithmic decisions are uncontestable. From a Conservation of Resources perspective, these safeguards help preserve resources such as certainty, control, confidence, and emotional security (Hobfoll, 1989; Hobfoll et al., 2018). Reduced technostress should subsequently strengthen employees’ capacity to exercise independent judgement and maintain control over their work. Thus, human oversight may support autonomy primarily by alleviating the cognitive and emotional strain associated with algorithmic management. Accordingly:

*H11:* Technostress mediates the relationship between human-in-the-loop presence and employee autonomy.

**Figure 1:**
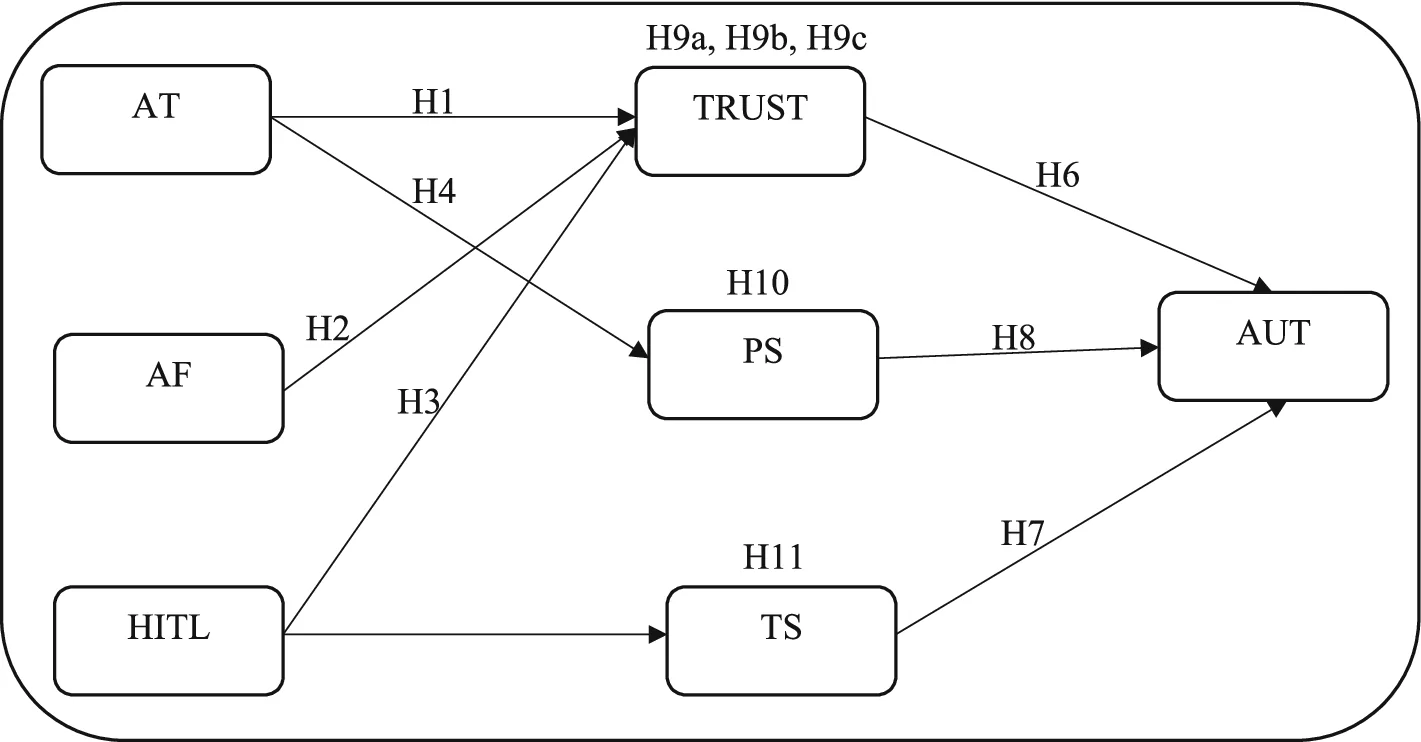
Proposed conceptual model.

Building on the theoretical arguments and hypotheses, Figure 1 presents the conceptual model of the study.

## 3 Methodology

### 3.1 Research design, study context, and sampling

The study adopted a quantitative, cross-sectional research design to examine the relationships among ethical AI-governance mechanisms, employee trust in AI, technostress, psychological safety, and employee autonomy. A post-positivist orientation guided the study because the proposed relationships were theoretically specified and empirically tested using employees’ perceptions of AI-enabled HRM practices. The design was appropriate for assessing the explanatory and predictive relationships among multiple latent constructs within an integrated structural model.

The study was conducted among employees working in organisations in India that had adopted AI-enabled HRM applications, including algorithm-supported recruitment, performance management, workforce scheduling, employee monitoring, and predictive workforce analytics. To qualify for participation, respondents were required to be full-time employees with direct experience of, or exposure to, AI-supported HR processes within their organisations. This criterion ensured that responses reflected actual workplace experiences rather than general opinions about artificial intelligence.

A purposive sampling technique was used to identify 290 employees who met the study’s eligibility criteria. The technique was appropriate because the study required respondents with specific knowledge and experience of AI-enabled HRM systems. Questionnaires were distributed directly to all 290 identified participants, and all were completed and returned, resulting in a 100% response rate and a final sample of 290 respondents.

Data were collected through a structured questionnaire administered electronically. The survey introduction explained the purpose of the study, the eligibility requirements, the voluntary nature of participation, and the confidentiality of responses. Participants were informed that no personally identifiable information would be collected and that they could discontinue participation at any stage. The questionnaire was pre-tested with academics and HR professionals to assess the clarity, comprehensibility, and contextual relevance of the items. Minor wording adjustments were made based on the feedback received before the main data collection.

The final questionnaire measured seven constructs using 23 items rated on a five-point Likert scale. The constructs comprised algorithmic transparency, perceived algorithmic fairness, human-in-the-loop presence, employee trust in AI, technostress, psychological safety, and employee autonomy. All measurement items were adapted from previously validated scales and contextualised to reflect employees’ experiences with AI-enabled HRM systems.

### 3.2 Measurement of constructs

The study measured seven reflective constructs using 23 items adapted from established scales and contextualised to AI-enabled HRM. Algorithmic transparency was measured using items adapted from Lee (2018), perceived algorithmic fairness from Binns et al. (2018) and Grgic-Hlaca et al. (2018), human-in-the-loop presence from Cummings (2004) and Amershi et al. (2019), employee trust in AI from Heerink et al. (2010), technostress from Tarafdar et al. (2007) and Ragu-Nathan et al. (2008), psychological safety from Edmondson (1999), and employee autonomy from Hackman and Oldham (1976). The questionnaire items are attached in Appendix A.

All items were assessed on a five-point Likert scale ranging from 1 = strongly disagree to 5 = strongly agree. The questionnaire was reviewed by academics and HR professionals for clarity, relevance, and comprehensibility, after which minor wording adjustments were made before final administration.

### 3.3 Data analysis

The data was analysed using partial least squares structural equation modelling (PLS-SEM). The main reason for choosing this approach was its ability to estimate measurement and structural relationships among multiple latent constructs while controlling for measurement error (Hair et al., 2019). PLS-SEM is robust with non-normal data distribution and moderate sample sizes which is common in organizational survey research (Hair et al., 2019). Secondly, the exploratory-predictive approach of the study is an inherent fit with the focus of PLS-SEM on maximum explained variance in endogenous constructs (*R*^2^) and not on reproducing the observed covariance matrix, as in CB-SEM. Thirdly, the proposed model includes both mediation chains and constructs with relatively small number of indicators (3–4 items per construct), which is the condition in which PLS-SEM has better statistical power and shows better convergence behaviour. Fourth, PLS-SEM is gaining popularity as an appropriate technique for theory building in the field of management and HRM research (Richter et al., 2016).

The significance tests were performed using the “bootstrapping” method with 5,000 subsamples and two-tailed testing, as recommended by Hair et al. (2019) for PLS-SEM applications. The common method bias assessment is provided. Since the study was cross-sectional and single source, the potential for common method bias (CMB) was evaluated with Harman’s single factor test as suggested by Podsakoff et al., (2003). All 23 items were entered simultaneously in an unrotated exploratory factor analysis and the first factor accounted for 27.3% of the variance, which is still far away from 50%, suggesting that common method bias does not seriously threaten the validity of the findings. Additionally, procedural remedies were employed during the design of the survey, such as distinguishing between predictor and criterion items in the different sections of the survey, using different scale anchors on the different items on the survey, and ensuring that the survey was anonymous to the respondents to reduce social desirability bias (Podsakoff et al., 2012).

## 4 Results

### 4.1 Measurement model assessment

Table 1 assessed indicator reliability, internal consistency reliability, convergent validity, and discriminant validity. Indicator reliability was evaluated through outer loadings, where all item loadings ranged from 0.776 to 0.841, exceeding the recommended threshold of 0.70 (Hair et al., 2019). Internal consistency reliability was assessed using composite reliability (CR) and Cronbach’s alpha (*α*). CR values ranged from 0.835 to 0.872, while Cronbach’s alpha values ranged from 0.725 to 0.819, both exceeding the recommended threshold of 0.70 (Nunnally, 1978). These findings confirm satisfactory internal consistency across all constructs. Convergent validity was examined using average variance extracted (AVE). AVE values ranged between 0.627 and 0.672, exceeding the minimum criterion of 0.50 (Fornell and Larcker, 1981), thereby confirming that each construct explains more than half of the variance in its indicators.

**Table 1:** Measurement model assessment.

Construct/item	Outer loading (*λ*)	AVE	CR	*α*	√AVE	Fornell–Larcker criterion
Algorithmic Transparency (AT)		0.631	0.872	0.812	0.794	Supported
AT1	0.782					
AT2	0.798					
AT3	0.776					
AT4	0.804					
Perceived Algorithmic Fairness (AF)		0.645	0.845	0.761	0.803	Supported
AF1	0.823					
AF2	0.801					
AF3	0.789					
Human-in-the-Loop Presence (HITL)		0.631	0.872	0.819	0.794	Supported
HITL1	0.791					
HITL2	0.808					
HITL3	0.779					
HITL4	0.797					
Employee Trust in AI (TRUST)		0.672	0.860	0.780	0.820	Supported
TR1	0.841					
TR2	0.822					
TR3	0.797					
Technostress (TS)		0.627	0.835	0.725	0.792	Supported
TS1	0.789					
TS2	0.801					
TS3	0.779					
Psychological Safety (PS)		0.660	0.853	0.758	0.812	Supported
PS1	0.831					
PS2	0.809					
PS3	0.794					
Employee Autonomy (AUT)		0.648	0.846	0.750	0.805	Supported
AUT1	0.812					
AUT2	0.799					
AUT3	0.804					

Discriminant validity was evaluated using the Fornell-Larcker criterion. The square root of AVE for each construct was greater than its inter-construct correlations, satisfying the Fornell-Larcker criterion (Henseler et al., 2015). Collectively, these results establish adequate discriminant validity for the measurement model.

To ensure that the path estimates were not distorted by multicollinearity between the predictor constructs, collinearity diagnostics was performed prior to examining the structural relationships through the use of variance inflation factor (VIF) values in Table 2. The inner VIF values were between 1.69 and 2.87, values that are far lower than the recommended value of 3.3 (Hair et al., 2019). The results here demonstrate that there are no multicollinearity concerns in the structural model.

**Table 2:** Variance inflation factor (VIF).

Endogenous construct	Predictor construct	VIF
TRUST	AT	2.14
TRUST	AF	2.32
TRUST	HITL	1.98
PS	AT	1.76
TS	HITL	1.69
AUT	TRUST	2.87
AUT	TS	2.11
AUT	PS	2.43

### 4.2 Structural model assessment and mediation analysis

After satisfactory quality of the measurement model was confirmed, the structural model was assessed to test the significance, direction, and magnitude of the hypothesized relationships as well as the model’s explanatory and predictive power. Following the two-stage PLS-SEM procedure suggested by Hair et al. (2019), the inner model was tested by generating bootstrapped subsample results of 5,000 iterations, with BCa (bias-corrected accelerated) confidence intervals under two-tailed testing. The results of the hypothesized direct structural relationships were statistically significant at the *p* < 0.001 level, as shown in Table 3. The strongest direct effect on the dependent variables was TRUST in AI (*β* = 0.389, t = 7.118); AUT (*β* = 0.341); and TS (*β* = −0.287). AT, AF, and HILP were significant factors in increasing TRUST in AI, and HILP was also found to decrease TS. Effect size analysis showed that the practical effects were small to moderate, with the greatest practical effect being the TRUST → AUT relationship (*f*^2^ = 0.151).

**Table 3:** Structural model assessment, mediation analysis, hypotheses.

Relationship/Construct	*β*	*t*-value	*p*-value	*f* ^2^	95% BCa CI	*R* ^2^	Adjusted *R*^2^	*Q* ^2^	Decision
Direct structural paths									
H1: AT → TRUST	0.312	5.841	<0.001	0.097		0.412	0.382	0.671	Supported
H2: AF → TRUST	0.284	5.219	<0.001	0.081		0.412	0.382	0.671	Supported
H3: HITL → TRUST	0.261	4.987	<0.001	0.068		0.412	0.382	0.671	Supported
H4: AT → PS	0.298	5.412	<0.001	0.089		0.341	0.315	0.612	Supported
H5: HITL → TS	−0.243	4.621	<0.001	0.059		0.287	0.261	0.531	Supported
H6: TRUST → AUT	0.389	7.118	<0.001	0.151		0.498	0.471	0.721	Supported
H7: TS → AUT	−0.287	5.014	<0.001	0.082		0.498	0.471	0.721	Supported
H8: PS → AUT	0.341	6.203	<0.001	0.116		0.498	0.471	0.721	Supported
Indirect (mediated) effects									
H9a: AT → TRUST → AUT	0.121	3.412	<0.001		[0.051, 0.193]				Supported
H9b: AF → TRUST → AUT	0.111	3.198	0.001		[0.043, 0.181]				Supported
H9c: HITL → TRUST → AUT	0.102	2.987	0.003		[0.035, 0.172]				Supported
H10: AT → PS → AUT	0.102	2.914	0.004		[0.034, 0.172]				Supported
H11: HITL → TS → AUT	0.070	2.641	0.008		[0.018, 0.124]				Supported

The mediation analyses also showed that the impact of AT, AF and HILP were partially mediated by TRUST in AI on AUT. Additionally, AT was partially mediated by PS in relation to AUT. Conversely, the connection between HILP and AUT was completely mediated by TS, suggesting that the role of human oversight in enhancing employee autonomy is primarily through its ability to lessen the stress caused by AI. The coefficients of determination (*R*^2^) ranged from moderate to substantial, explaining variance for endogenous constructs, with AUT having the highest explained variance of *R*^2^ = 0.498. All *Q*^2^ values were positive through blindfolding, reflecting good predictive performance. The coefficients of determination indicated moderate explanatory power, with the model explaining 41.2% of the variance in employee trust in AI, 34.1% in psychological safety, 28.7% in technostress, and 49.8% in employee autonomy. Furthermore, all *Q*^2^ values exceeded zero, indicating predictive relevance for the endogenous constructs (*Q*^2^ = 0.721).

## 5 Discussion and implications

The findings demonstrate that employee autonomy in AI-enabled workplaces depends significantly on how algorithmic systems are governed and psychologically experienced. Algorithmic transparency, perceived algorithmic fairness, and human-in-the-loop presence significantly strengthened employee trust in AI, jointly explaining 41.2% of its variance. Among these mechanisms, transparency produced the strongest effect, suggesting that employees’ confidence in AI begins primarily with their ability to understand how algorithm-supported HR decisions are generated. This supports earlier research showing that opacity creates informational asymmetry and uncertainty, whereas understandable explanations enhance the perceived predictability and legitimacy of algorithmic decisions (Lee, 2018; Wieringa, 2020; Bujold et al., 2024).

Perceived algorithmic fairness also strengthened trust in AI. Employees therefore evaluate algorithmic systems not only by their technical accuracy but also by whether their procedures and outcomes are considered unbiased, consistent, and appropriate. This finding is consistent with Binns et al. (2018) and Grgic-Hlaca et al. (2018), who showed that fairness judgements influence individuals’ reactions to algorithmic decisions. It also reinforces the organisational justice argument that employees’ acceptance of decisions depends on both the outcomes received and the procedures through which they are produced (Greenberg, 1987). Human-in-the-loop presence similarly enhanced trust, indicating that employees place greater confidence in AI-supported decisions when accountable managers retain the authority to review, explain, or overturn algorithmic recommendations. This supports Amershi et al. (2019) and algorithm-aversion research suggesting that people are more comfortable with algorithmic systems when meaningful human intervention remains possible (Dietvorst et al., 2016).

Algorithmic transparency also improved psychological safety. Employees who understand the basis of AI-supported decisions appear more comfortable questioning, discussing, or challenging those decisions. Transparency therefore does more than enhance system comprehension; it provides an informational foundation for employee voice. This extends Edmondson’s (1999) psychological safety argument to algorithmically managed workplaces, where employees may need to report inaccurate data, identify bias, or request reconsideration of automated outcomes. However, transparency is likely to support voice only where organisations treat questions about algorithmic decisions as legitimate rather than as resistance to technological change.

Human-in-the-loop presence reduced technostress, supporting the view that human oversight functions as both a technical safeguard and a psychological resource. Employees experience less strain when they can seek explanations, correct inaccurate information, and appeal decisions rather than being subjected to uncontestable algorithmic control. This finding aligns with Conservation of Resources Theory, as meaningful oversight preserves certainty, confidence, and perceived control that might otherwise be depleted by opaque monitoring and automated evaluation (Hobfoll, 1989; Hobfoll et al., 2018).

Trust in AI emerged as the strongest predictor of employee autonomy, followed by psychological safety, while technostress reduced autonomy. These findings show that employees retain greater agency when they regard AI systems as dependable, feel safe challenging their outputs, and possess sufficient cognitive and emotional resources to exercise independent judgement. They support the duality perspective that algorithmic management may either enable or constrain autonomy depending on its design and governance (Kellogg et al., 2020; Meijerink and Bondarouk, 2023).

The mediation results further clarify these mechanisms. Trust transmitted the effects of transparency, fairness, and human oversight to autonomy, demonstrating that governance mechanisms preserve agency partly by building confidence in AI. Psychological safety mediated the transparency–autonomy relationship, showing that information becomes empowering when employees feel safe using it to question decisions. Technostress mediated the relationship between human oversight and autonomy, indicating that human involvement preserves agency by reducing uncertainty and psychological strain. Overall, ethical AI governance supports employee autonomy through three complementary pathways: relational confidence through trust, interpersonal security through psychological safety, and resource preservation through reduced technostress.

### 5.1 Theoretical contributions

This study makes three theoretical contributions to the literature on algorithmic management, AI-enabled HRM, and employee autonomy. First, it develops an integrated explanation of how ethical AI-governance mechanisms shape employee autonomy. Existing research has commonly examined algorithmic transparency, fairness, human oversight, trust, technostress, psychological safety, and autonomy in separate streams. By bringing these constructs together, the study shows that the autonomy consequences of AI-enabled HRM cannot be understood from the technical characteristics of algorithmic systems alone. Rather, employee autonomy depends on how employees interpret the transparency, fairness, and accountability of those systems.

Second, the study identifies three distinct psychological pathways through which ethical AI governance influences autonomy. Trust in AI represents a relational pathway through which transparency, fairness, and human oversight strengthen employees’ confidence in algorithmic systems. Psychological safety represents an interpersonal pathway through which transparency enables employees to question, challenge, and seek reconsideration of algorithmic decisions. Technostress represents a resource-depletion pathway through which weak or absent human oversight can undermine employees’ capacity for autonomous action. By distinguishing these mechanisms, the study extends existing research beyond the broad argument that algorithmic management either enables or constrains autonomy and explains the processes through which these outcomes emerge.

Third, the study extends social exchange theory and conservation of resources theory to the context of AI-enabled HRM. Social exchange theory is extended by showing that employees interpret algorithmic governance practices as organisational signals of openness, justice, and accountability, which subsequently shape trust and psychological safety. Conservation of Resources Theory is extended by demonstrating that meaningful human oversight preserves employees’ psychological resources by reducing uncertainty, loss of control, and technostress. Together, these theories provide a more complete explanation of employee autonomy in algorithmically managed workplaces by linking relational confidence, interpersonal security, and resource preservation. The study therefore positions employee autonomy as an important theoretical criterion for evaluating responsible AI-enabled HRM, alongside more commonly examined outcomes such as acceptance, trust, and performance.

### 5.2 Practical implications

The findings offer several practical implications for organisations adopting AI-enabled HRM systems. First, organisations should treat algorithmic transparency as a core governance practice rather than a technical disclosure exercise. Employees should receive clear and understandable explanations of the data used, the criteria applied, and the role of AI in decisions affecting recruitment, performance evaluation, scheduling, promotion, and compensation. Transparency should enable employees to understand and question decisions, not merely provide complex technical information that remains inaccessible to non-specialists.

Second, organisations should establish mechanisms for monitoring and improving perceived algorithmic fairness. This requires regular fairness audits, review of training data, testing for inconsistent outcomes across employee groups, and clear procedures for correcting biased or inaccurate decisions. Because employees judge fairness through both processes and outcomes, organisations should also communicate why particular criteria are used and how employees can challenge decisions they consider inappropriate.

Third, human-in-the-loop oversight should be meaningful rather than symbolic. Managers responsible for reviewing AI-generated recommendations should possess sufficient knowledge, authority, and access to relevant information to revise or overturn system outputs. Employees should also know who remains accountable for final decisions and how to request human review. This is especially important for high-stakes decisions involving performance ratings, promotion, discipline, compensation, and termination.

The findings further indicate that organisations must address the psychological conditions surrounding AI use. Transparent communication should be accompanied by a psychologically safe environment in which employees can raise concerns, report errors, and question algorithmic outcomes without fear of retaliation. Managers should actively encourage such voice and treat employee feedback as an important source of information for improving AI systems.

Finally, organisations should monitor and reduce technostress associated with algorithmic management. AI literacy programmes, user training, accessible support, gradual implementation, and clear appeal processes can reduce uncertainty and perceived loss of control. By combining transparency, fairness safeguards, accountable human oversight, psychological safety, and employee support, organisations can use AI to improve efficiency without undermining autonomy, trust, and employee well-being.

## 6 Limitations and future research

As with all studies, there are boundaries to this one. Because of the cross-sectional design, caution should be used in making causal inferences; although the theoretical foundation of social exchange theory and conservation of resources theory is sound, reverse causality is not necessarily eliminated. Future research could benefit from either a longitudinal or experimental design to follow the changes in trust, technostress, and autonomy over time as AI usage intensifies. Self-reported data is also a limitation. Multi-source data, behavioural observations or objective organisational data could be utilised in future research. However, the Indian organizational context restricts the generalizability of the findings, as cultural norms, the nature of work, and attitudes toward automation are very different in other cultures. The model also might be extended to incorporate other factors such as AI literacy, leadership style, and job complexity, alongside outcomes such as innovation behaviour, turnover intention, and employee engagement. Moving forward, it could be interesting to investigate if transparency, fairness, and human oversight can be integrated into one higher-order ethical AI governance concept, possibly providing a purer and more complete view of how AI systems are influencing the digital workplace.

## 7 Conclusion

This study examined how algorithmic transparency, perceived algorithmic fairness, and human-in-the-loop presence shape employee autonomy through trust in AI, psychological safety, and technostress. The findings show that ethical AI governance strengthens autonomy when employees understand algorithmic decisions, perceive them as fair, retain access to accountable human judgement, and feel safe questioning system outputs. Trust in AI and psychological safety enhanced autonomy, whereas technostress reduced it.

The study demonstrates that responsible AI-enabled HRM is not achieved through technical efficiency alone. Organisations must design and govern AI systems in ways that preserve employee voice, discretion, psychological security, and meaningful human accountability. Ethical AI governance is therefore central to creating digital workplaces in which technological advancement and employee autonomy can coexist.

## Data Availability

The raw data supporting the conclusions of this article will be made available by the authors, without undue reservation.

## Appendix A

**Table A1:** Questionnaire items.

Construct	Code	Questionnaire item
Algorithmic transparency	AT1	I understand how AI makes HR decisions.
	AT2	AI decision processes are clearly explained to me.
	AT3	I can question AI-based decisions.
	AT4	The organisation provides clear documentation on the AI algorithms used in HR processes.
Perceived algorithmic fairness	AF1	AI-based HR decisions are unbiased.
	AF2	AI-based HR systems treat employees equally.
	AF3	AI-based HR decisions are applied consistently.
Human-in-the-loop presence	HITL1	Managers review AI-generated HR decisions.
	HITL2	Human decision-makers can override AI-generated decisions.
	HITL3	AI-supported HR decisions are not made entirely autonomously.
	HITL4	There is regular human oversight of AI-driven HR processes.
Employee trust in AI	TRUST1	I trust the use of AI in HR processes.
	TRUST2	AI-supported HR decisions are reliable.
	TRUST3	AI-supported HR systems act in employees’ best interests.
Technostress	TS1	The use of AI systems increases my work-related stress.
	TS2	I feel overwhelmed by AI-based monitoring.
	TS3	The use of AI reduces my sense of control over my work.
Psychological safety	PS1	I feel safe expressing concerns about AI-supported HR decisions.
	PS2	I can challenge AI-generated decisions without fear of negative consequences.
	PS3	My organisation supports open discussion about the use of AI.
Employee autonomy	AUT1	I have control over decisions concerning my work.
	AUT2	The use of AI does not restrict my independence at work.
	AUT3	I can make work-related decisions freely despite the use of AI.

All items were measured using a five-point Likert scale ranging from 1 = strongly disagree to 5 = strongly agree. The items were adapted from established measures and contextualised to AI-enabled HRM.
