# Supplementary material for: Governing algorithms, empowering people: how ethical AI oversight shapes trust, technostress, and employee autonomy in AI-enabled HRM
Source: Front Artif Intell. 2026 Aug 31;9:1911545. doi: 10.3389/frai.2026.1911545 (PMC13572705; doi:10.3389/frai.2026.1911545)
Supplement: Supplementary file 1 [file Data_Sheet_1.DOCX]

*Source-Authors own compilation*

***Note. AT = Algorithmic Transparency; AF = Perceived Algorithmic Fairness; HITL = Human-in-the-Loop Presence; TRUST = Employee Trust in AI; TS = Technostress; PS = Psychological Safety; AUT = Employee Autonomy. Solid blue arrows indicate positive (enabling) hypothesized paths; dashed arrows indicate negative (inhibitory) hypothesized paths. H1–H8 denote the eight directional research hypotheses. The model is grounded in Social Exchange Theory (Blau, 1964) and the Conservation of Resources framework (Hobfoll, 1989). The central column (TRUST, TS, PS) represents the three mediating mechanisms through which governance antecedents are hypothesized to influence employee autonomy.***
